# Screening the Drug-Drug Interactions Between Antimicrobials and Other Prescribed Medications Using Google Bard and Lexicomp® Online™ Database

**DOI:** 10.7759/cureus.44961

**Published:** 2023-09-09

**Authors:** Dilveen M Sulaiman, Suhail S Shaba, Hind B Almufty, Asmaa M Sulaiman, Muayad A Merza

**Affiliations:** 1 Department of Pharmacology, College of Pharmacy, University of Duhok, Duhok, IRQ; 2 Department of Pharmaceutics, College of Pharmacy, University of Duhok, Duhok, IRQ; 3 Department of Clinical Pharmacy, College of Pharmacy, University of Duhok, Duhok, IRQ; 4 Department of Clinical Pharmacy, Azadi Teaching Hospital, Duhok, IRQ; 5 Department of Internal Medicine, Azadi Teaching Hospital, Duhok, IRQ

**Keywords:** ai and robotics in healthcare, antimicrobial agent, lexicomp, google bard, drug-drug interactions

## Abstract

Aim

This study aimed to critically appraise the drug-drug interaction (DDI) screening performance of Google Bard (Google AI, Mountain View, California, United States) by comparing it with the authorized Lexicomp® Online™ database (Wolters Kluwer Health, Philadelphia, Pennsylvania, United States).

Methods

This cross-sectional study was conducted between April 2023 and August 2023, and enrolled 414 prescriptions that had been collected randomly between April 2023 and June 2023. These prescriptions were processed individually by Lexicomp online and Google Bard to screen for DDIs between antimicrobials and other prescribed medications.

Results

The total number of DDIs based on Lexicomp and Google Bard were 90 and 68, respectively. Cohen's Kappa (κ) values showed that there was a nil to slight agreement between Lexicomp and Google Bard regarding the DDI risk rating (κ=0.01). Regarding the severity rate, there was a slight agreement between them (κ=0.02), but in terms of reliability rate, there was no agreement (κ =-0.02).

Conclusion

This study unveiled differences between Lexicomp and Google Bard regarding their DDI identification, severity rating, and reliability rates. It is fundamental to consider that both tools have their strengths and weaknesses and, therefore, should not be individually depended on for final clinical decisions. However, Lexicomp can be considered authoritative in screening DDIs, but Google Bard currently lacks the necessary precision and reliability for conducting such screenings.

## Introduction

A drug-drug interaction (DDI) is a phenomenon in which the effects of a drug are altered by the presence of another drug, which can result in a change in the pharmacokinetic and/or pharmacodynamic profile of a drug that may enhance or reduce the drug effect or cause whole new effects to appear [1].

The total clinically manifested DDI prevalence worldwide is 9.2%, and approximately 1% of all hospital admission is caused by DDIs [2,3]. In hospital settings, undesirable DDIs are a fundamental health concern as they link with unwanted outcomes like longer hospital stays and clinical deterioration (changes in the respiratory rate, oxygen saturation, blood pressure, heart rate, temperature, and consciousness), and sometimes result in mortality [4].

Antimicrobial-drug interaction incidence is having a greater extent as these agents are used widely in both inpatients and outpatients [5]. In Iraq, antibiotics are the medicines most commonly associated with prescribing errors because healthcare providers prescribe antibiotics without taking into account their interactions with chronic medications [6]. In addition to that, these agents are among the most freely and excessively dispensed medicines by community pharmacies without prescriptions [7].

Several databases have been created to aid prescribers in detecting DDIs by enhancing patient safety and reducing adverse effects [8]. These databases have been developed exponentially over the past decades, coinciding with the artificial intelligence (AI) revolution. Drugs.com (Auckland, New Zealand), Lexicomp® (Wolters Kluwer Health, Philadelphia, Pennsylvania, United States), and epocrates® (Watertown, Massachusetts, United States) are among the most reliable and precise DDI screening programs that healthcare professionals rely on to identify potential interactions [9,10].

Lexicomp is a DDI database for healthcare providers and requires a subscription for access. It has been developed in various platforms, such as mobile applications, online, or desktop software. The risks of DDI by this platform are rated from A (no known interaction) to X (avoid combination) [11].

Google Bard is a large language model from Google AI (Mountain View, California, United States), run by Language Model for Dialogue Applications (LaMDA), trained on a massive dataset; it is similar to OpenAI's Chat Generative Pre-Trained Transformer (ChatGPT). LaMDA is a transformer-based neural language model pre-trained on online chat data [12]. Google Bard has been shown to be effective in a variety of scientific applications but has yet to be for DDI evaluation.

To our knowledge, there have been no studies evaluating the possible antimicrobial DDIs in various healthcare sets in Iraq and the Kurdistan region using AI software. Thus, this study aimed to critically appraise the DDI screening performance of Google Bard by comparing it with the authorized Lexicomp Online database. 

## Materials and methods

Study design and sampling

This cross-sectional observational study was performed between April 2023 and August 2023, and samples of medical prescriptions were randomly collected from various community and outpatient hospital pharmacies in the Duhok province, Iraq.

The prescriptions were sorted according to the number of medications and antimicrobials, source of prescription, either dispensed in hospital or community pharmacies, and the patient demographic data, gender, and age. A Microsoft Excel™ spreadsheet (Microsoft Corporation, Redmond, Washington, United States) was used to input, manage, and manipulate data in tabular format.

Study tools and procedure

Each medical prescription was processed individually by the Lexicomp Online database and Google Bard to screen for potential interactions between the antimicrobial drugs and other prescribed medications. Lexicomp was chosen to serve as a reference database as, based on previously published literature suggestions, it is thought to be one of the best resources in the field [13,14]. The assessment results then have been tabulated in an Excel sheet to risk, severity, and reliability ratings associated with each interaction.

DDI risk, severity, and reliability stratification

The interaction analysis screen in the Lexicomp Online database usually provides a summary of interactions; moreover, Google Bard provided an analogous summary based on our request during data processing, including an assigned risk rating (A, B, C, D, or X) that appears next to each DDI. The assigned alphabetic symbols correspond to distinct degrees of urgency in addressing the identified interactions. The description of each risk rating is given in Table 1 [15].

**Table 1:** Drug-drug interaction risk rating

Risk Rating	Action	Description
A	No Known Interaction	Data do not provide evidence of pharmacodynamics or pharmacokinetic interactions between the listed medications.
B	No Action Required	Data indicate that the listed medications have the potential to interact with one another, but no evidence of significant clinical concerns from simultaneous use.
C	Monitor Therapy	Data show that a clinically significant interaction may occur between the listed medications. The advantages of using these two medication concurrently usually outweigh the risks.
D	Modify Therapy	Data indicate clinically significant interactions between the listed medications. A patient-specific assessment should be performed to determine whether the advantages of concurrent therapy outweigh the risks, and specific measures should be considered to get the benefits and/or reduce the harmful effects of using the medications together.
X	Avoid Combination	Data indicate clinically significant interactions between the listed medications. The potential risks of the simultaneous use of these medications generally outweigh the benefits. Their concomitant use is generally contraindicated.

Table 2 displays the severity levels for the particular interaction; severity rating serves to assess the interaction's medical risk [15].

**Table 2:** Drug-drug interaction severity rating

Severity Rating	Description
Major	The interaction could be fatal or cause long-lasting or irreversible harm.
Moderate	The interaction could result worsening of the condition; could need additional care.
Minor	The interaction could result in uncomfortable symptoms but not medically harmful.

Reliability rating indicates the quantity and nature of documentation for the interaction: poor, fair, good, or excellent [16].

Inclusion and exclusion criteria

Medical prescriptions that contained at least one antimicrobial agent and one other medication dispensed in hospital and community pharmacies between April 2023 and June 2023 were included in the study. While prescriptions that lacked antimicrobial agents, and those that contained medications not found in Lexicomp Online or could not be processed by Google Bard were excluded from the study.

Data analysis

Data were analyzed descriptively using the statistical computing programming language R version 4.2.2 and presented as frequencies and percentages. We calculate Cohen's Kappa value a measure of the agreement that ranges from (-1 to +1) with their corresponding standard errors; to evaluate the accuracy of Google Bard. P values of 0.05 were considered statistically significant.

Cohen proposed the following interpretation for the Kappa findings: values less than or equal to zero were indicative of no agreement, while values ranging from 0.01-0.20 indicated a nil to slight agreement, 0.21-0.40 represented fair agreement, 0.41- 0.60 indicated moderate agreement, 0.61-0.80 indicated substantial agreement and 0.81-1.00 denoted perfect agreement [17].

Scientific and ethical approval

The study was reviewed and approved by the Scientific and Ethics Committee of the College of Pharmacy at the University of Duhok, Iraq, on March 7, 2023 (Reference No. 642). The study only involved the record of prescriptions and DDIs of medications dispensed.

## Results

Demographic data

The study enrolled 414 medical prescriptions filled by about 190 prescribers. The majority (243) were collected from community pharmacies, and the remaining (171) were from hospital outpatient ones. Of the prescriptions, 44.2% were written for females and 24.63% were for patients aged below 40 years. The total number of medications prescribed was 1476 and the average number of medicines per prescription was 3.6; 40.78% of medications prescribed were antimicrobial agents and their average number per prescription was 1.5 (Table 3). 

**Table 3:** Demographic data

Variables	Number	Percentage
Gender		
Male	113	27.3
Female	183	44.2
Missing	118	28.5
Age		
≤39	102	24.64
≥40	34	8.21
Missing	278	67.15
Pharmacies		
Community	243	58.7
Hospital	171	41.30
Medications		
Antimicrobial agents	602	40.79
Other medications	874	59.21

Risk, severity, and reliability rating of DDIs

Based on Lexicomp, 324 prescriptions showed no known interactions, whereas Google Bard identified 346 prescriptions with no known interactions. The total number of DDIs based on Lexicomp and Google Bard were 90 and 68, respectively. The majority of the DDIs were in the moderate severity level in Lexicomp (52.22%), while the major severity level accounted for the lowest percentage in Google Bard (2%) (Table 4).

**Table 4:** Drug-drug interaction risk, severity, and reliability rating frequencies and percentages by Lexicomp and Google Bard Lexicomp® Online™, Wolters Kluwer Health, Philadelphia, Pennsylvania, United States Google Bard, Google AI, Mountain View, California, United States

	Lexicomp		Google Bard	
	Number	Percentage	Number	Percentage
Risk Rating				
A	324	78.26	346	83.57
B	26	6.28	58	14.01
C	53	12.8	8	1.93
D	11	2.66	0	0
X	0	0	2	0.48
Total	414	100	414	100
Severity Rating				
Minor	27	30	57	83.82
Moderate	47	52.22	9	13.24
Major	16	17.78	2	2.94
Total	90	100	68	100
Reliability Rate				
Poor	4	4.44	1	1.47
Fair	56	62.22	19	27.94
Good	18	20	47	69.12
Excellent	12	13.33	1	1.47
Total	90	100	68	100

Agreement between Lexicomp and Google Bard

An inter-rater reliability analysis was performed between the risk rating in Lexicomp and Google Bard, and Cohen's Kappa was calculated, which is a measure of the agreement. It showed that there was a nil to slight agreement between them regarding DDI risk rating, with a κ value = 0.01. Regarding the severity rate, Cohen's Kappa showed a slight agreement between the severity rating in Lexicomp and Google Bard, with κ= 0.02. However, in terms of reliability rate, there was no agreement between Lexicomp and Google Bard, with κ value of -0.02 (Table 5).

**Table 5:** Agreement between Lexicomp and Google Bard Lexicomp® Online™, Wolters Kluwer Health, Philadelphia, Pennsylvania, United States Google Bard, Google AI, Mountain View, California, United States

Variables	Cohen's Kappa	95% CI	P value
Risk rate	0.01	(-0.12-0.15)	0.871
Severity rate	0.02	(-0.12-0.15)	0.802
Reliability rate	-0.02	(-0.15-0.12)	0.199

## Discussion

DDIs are of grave concern in healthcare settings, remarkably when antimicrobial agents are conjointly prescribed with other medications. In this study, DDIs between antimicrobial agents and other prescribed medications were investigated not only in governmental pharmacy settings but also in private ones. To our knowledge, this is plausibly the first study comparing Google Bard with Lexicomp globally.

Out of the 1476 recorded medications, 40.79% were antimicrobial agents. The substantial proportion of anti-infective medications in this study indicates their significance in managing infectious diseases. Meanwhile, a notable relationship exists between the rates of antibiotic consumption and the escalation of antibiotic resistance. Another urgent global concern is the emergence of antibiotic-resistant strains. As reported by the United States Centers for Disease Control and Prevention (CDC), antimicrobial resistance was responsible for causing the deaths of at least 1.27 million individuals worldwide and was associated with nearly 5 million fatalities in 2019 [18]. Furthermore, we found that from an average of 3.6 medications, approximately 1.5 was the average number of antimicrobials per prescription. This finding weighs a necessity to attentively assess potential DDIs while prescribing anti-infective agents to accomplish successful treatment outcomes.

In our study, the Lexicomp and Google Bard comparison in pinpointing DDIs and evaluating severity and reliability rates led to interesting findings. To demonstrate, Google Bard detected 68 prescriptions having at least one DDI, while Lexicomp detected a higher rate with a number of 90; therefore, Lexicomp reported approximately 1.3-fold higher DDIs.

Such a difference in the number of detected DDIs between Lexicomp and Google Bard is attributed to their variant databases, algorithms, and data sources. It should be noted that Lexicomp is a trusted and broadly used tool by the healthcare community. Lexicomp constitutes a comprehension of a wide drug interaction database variety, which may explain its efficiency in detecting DDIs [11]. To elucidate, a study comparing five soft databases by Kheshti et al. found that Lexi-Interact carries the best performance in detecting DDIs [10]. Even though Google Bard is created by a technology rich in data, it might spot DDIs differently, leading to its lower detection rate. Lexicomp and Google Bard met a slight agreement regarding risk rating. Conversely, a moderate consistency between Lexicomp and Micromedex (Merative L.P., Ann Arbor, Michigan, United States) was found in a study by Lie et al. [19]. Moreover, a study conducted in Qatar comparing Lexicomp and Micromedex found a moderate strength of agreement between the two databases while identifying potential DDIs [20].

Google Bard and Lexicomp appeared to have differences in their classifications with DDI severity ratings. While Lexicomp relayed higher percentages of moderate severity DDIs (52.22%), Google Bard reported lower (13.24%). On the other hand, Google Bard detected higher percentages of minor severity DDIs (83.82%) compared to Lexicomp (30%). This emphasizes Google Bard’s tendency to classify more interactions as minor severity, whereas Lexicomp behaved conversely. Furthermore, a slight agreement with respect to severity rating was noticed between the two systems. The reported disagreement considering the severity rating in this study corresponds to other literature [21].

This study found no agreement with respect to the reliability rating between Google Bard and Lexicomp. The variant reliability rates could be assigned to factors such as differences in databases, information sources, and algorithms. Moreover, divergences in available evidence concerning different drug combinations may present differences in reliability rates.

The main limitations of this study could be due to its small sample size, focus only on screening DDI between antimicrobials and other prescribed medications, and its lack of real clinical DDI of potential DDI investigations because although Lexicomp and Google Bard serve as valuable drug information resources but should not be considered substitutes for the professional judgment of healthcare providers. This is mainly due to the lack of resources considering this is the first research comparing Google Bard and Lexicomp globally to our knowledge. The scarcity of resources due to the lack of studies on this topic globally has hindered us from undertaking a precise comparison between other studies regarding Google Bard and Lexicomp.

## Conclusions

This study unveiled differences between Lexicomp and Google Bard regarding their DDI identification, risk, severity, and reliability rates. It is fundamental to consider that both tools have their strengths and weaknesses and, therefore, should not be individually depended on for final clinical decisions. However, Lexicomp is authoritative in screening DDIs, but Google Bard currently lacks the necessary precision and reliability for conducting such screenings. Healthcare professionals should use their clinical judgment, take additional resources into account, and note patient-specific elements while supervising drug interactions. This is the most optimal way to confirm a patient’s safety and enhance treatment outcomes. Further studies with larger sample sizes, especially based on Google Bard, are guaranteed to boost drug interaction screening tools’ reliability and precision.

## References

[1] Mousavi S, Ghanbari G (2017). Potential drug-drug interactions among hospitalized patients in a developing country. Caspian J Intern Med.

[2] Gonzaga de Andrade Santos TN, Mendonça da Cruz Macieira G, Cardoso Sodré Alves BM (2020). Prevalence of clinically manifested drug interactions in hospitalized patients: a systematic review and meta-analysis. PLoS One.

[3] Pirmohamed M, James S, Meakin S (2004). Adverse drug reactions as cause of admission to hospital: prospective analysis of 18 820 patients. BMJ.

[4] Lenssen R, Heidenreich A, Schulz JB, Trautwein C, Fitzner C, Jaehde U, Eisert A (2016). Analysis of drug-related problems in three departments of a German University hospital. Int J Clin Pharm.

[5] Pai MP, Momary KM, Rodvold KA (2006). Antibiotic drug interactions. Med Clin North Am.

[6] Al-Jumaili AA, Jabri AM, Al-Rekabi MD, Abbood SK, Hussein AH (2016). Physician acceptance of pharmacist recommendations about medication prescribing errors in Iraqi hospitals. INNOVATIONS in Pharmacy.

[7] Almufty HB, Hayhat L, Abdal Abdal WG (2023). The magnitude of dispensing unprescribed antibiotics in community pharmacies in Duhok province; Kurdistan region of Iraq. J Contemp Med Sci.

[8] Zenziper Straichman Y, Kurnik D, Matok I (2017). Prescriber response to computerized drug alerts for electronic prescriptions among hospitalized patients. Int J Med Inform.

[9] Vonbach P, Dubied A, Krähenbühl S, Beer JH (2008). Evaluation of frequently used drug interaction screening programs. Pharm World Sci.

[10] Kheshti R, Aalipour M, Namazi S (2016). A comparison of five common drug-drug interaction software programs regarding accuracy and comprehensiveness. J Res Pharm Pract.

[11] (2023). Lexicomp: Evidence-Based Drug Referential Content. https://www.wolterskluwer.com/en/solutions/lexicomp.

[12] Rahaman MS, Ahsan MM, Anjum N, Rahman MN, Rahman MM (2023). The AI race is on! Google's Bard and OpenAI's ChatGPT head to head: an opinion article. SSRN.

[13] Koppen L, Phillips J, Papageorgiou R (2015). Analysis of reference sources used in drug-related Wikipedia articles. J Med Libr Assoc.

[14] Mountford CM, Lee T, de Lemos J, Loewen PS (2010). Quality and usability of common drug information databases. Can J Hosp Pharm.

[15] (2023). Lexicomp Drug Interactions Analysis. https://www.wolterskluwer.com/en/solutions/lexicomp/resources/lexicomp-user-academy-old/drug-interactions-analysis.

[16] Nusair MB, Al-Azzam SI, Arabyat RM, Amawi HA, Alzoubi KH, Rabah AA (2020). The prevalence and severity of potential drug-drug interactions among adult polypharmacy patients at outpatient clinics in Jordan. Saudi Pharm J.

[17] McHugh ML (2012). Interrater reliability: the kappa statistic. Biochem Med (Zagreb).

[18] Antibiotic Resistance Threats in the United States, 2019 2019 (2023). Antibiotic Resistance Threats in the United States, 2019.. 2019.

[19] Liu Y, Wang J, Gong H (2023). Prevalence and associated factors of drug-drug interactions in elderly outpatients in a tertiary care hospital: a cross-sectional study based on three databases. Ann Transl Med.

[20] Abbas A, Al-Shaibi S, Sankaralingam S, Awaisu A, Kattezhathu VS, Wongwiwatthananukit S, Owusu YB (2022). Determination of potential drug-drug interactions in prescription orders dispensed in a community pharmacy setting using Micromedex(®) and Lexicomp(®): a retrospective observational study. Int J Clin Pharm.

[21] Roblek T, Vaupotic T, Mrhar A, Lainscak M (2015). Drug-drug interaction software in clinical practice: a systematic review. Eur J Clin Pharmacol.

